# The Awareness of the International Veterinary Profession of Evidence-Based Veterinary Medicine and Preferred Methods of Training

**DOI:** 10.3390/vetsci4010015

**Published:** 2017-03-08

**Authors:** Selene J. Huntley, Rachel S. Dean, Marnie L. Brennan

**Affiliations:** Centre for Evidence-based Veterinary Medicine (CEVM), School of Veterinary Medicine and Science, University of Nottingham, Sutton Bonington Campus, Leicestershire LE12 5RD, UK; selene.huntley@nottingham.ac.uk (S.J.H.); rachel.dean@nottingham.ac.uk (R.S.D.)

**Keywords:** evidence-based veterinary medicine, evidence based veterinary medicine, EVM, EBVM, veterinarians, veterinary surgeons, veterinary education, continuing professional development, CPD, training

## Abstract

Evidence-based veterinary medicine (EVM) is an evolving discipline in veterinary medicine so it is important to periodically “benchmark” opinion about EVM across the profession. An international survey to assess veterinarians’ awareness of EVM was conducted. Veterinarians were surveyed via an online questionnaire (all countries) or a postal questionnaire (UK only). Participants were asked whether they had heard of EVM, where they had first heard the term, and their preferences of method for receiving continuing professional development (CPD). There were 6310 respondents, of which 4579 (72.5%) worked in the UK and 5384 (85.3%) were clinicians. Veterinarians that had heard of EVM (*n* = 5420, 85.9%) were most likely to be clinicians (OR = 4.00; 95% CI: 3.37, 4.75), respondents working in the UK (OR = 1.32; CI: 1.13, 1.54), or respondents with a postgraduate degree or qualification (OR = 1.77; CI: 1.51, 2.08). The most common sources from which respondents had heard of EVM were at vet school or university (*n* = 1207, 29.8%), via literature (peer-reviewed papers or other publications) (*n* = 1074, 26.5%), and via CPD courses (*n* = 564, 13.9%). Most respondents were interested in finding out more about EVM (*n* = 4256 of 6173, 69%). The preferred methods of CPD were day or evening seminars (*n* = 2992 of 6017, 49.7%), conferences (*n* = 1409, 23.4%), and online courses (*n* = 524, 8.7%), although the order of preference differed slightly between groups. There appears to be substantial awareness of EVM amongst veterinarians internationally. However, it appears that further training in EVM would be welcomed. Preferences on how CPD in general is received differs between groups, so this should be borne in mind by training providers when formulating a strategy for the dissemination of EVM training across the global profession.

## 1. Introduction

Evidence-based veterinary medicine (EVM) is defined as “the use of best relevant evidence in conjunction with clinical expertise to make the best possible decision about a veterinary patient. The circumstances of each patient, and the circumstances and values of the owner/carer, must also be considered when making an evidence-based decision” [1,2]. Frequently in veterinary practice, clinical decisions are made on the basis of what has been used previously by an individual clinician or, in unusual clinical cases, according to advice from colleagues, specialists, laboratories, or the internet, rather than journal articles in bibliographic databases such as PubMed [3]. Evidence-based veterinary medicine is an important approach in relation to maintaining objectivity in the interpretation of treatment outcomes, thus avoiding decisions based on selective recall and the overestimation of successes [4].

The development of EVM as a discipline is built on the fundamentals of evidence-based medicine (EBM) in the field of human healthcare, where the integration of clinical expertise with the best available clinical evidence from systematic research is an established but sometimes contentious method of assisting clinicians in decision-making for individual patients [5]. This process requires the development of skills such as efficient literature searching and appraisal [6]. However, the ability of medical practitioners to practice EBM may be hindered by access to bibliographic databases such as Medline and a lack of time [7,8]. In developing countries particularly, the adoption of EBM approaches in practice has been hampered by poorer access to resources [9], such as full open access articles and articles in languages other than English, as well as a paucity of teachers who are clinically trained in EBM methods [10].

For busy practitioners, the evidence-based process is aided by the availability of summaries of best available evidence, such as guidelines accessed via NICE [11] which are often derived from systematic reviews conducted by the Cochrane Collaboration [6,12]. However, similar to veterinary medicine, frequently there are often insufficient good quality studies to enable a systematic review for a clinical question to be conducted in the first place. To aid users in making sense of what evidence there is available, online evidence synthesis resources that provide evidence summaries were established in the late 1990s for medics [13] and in 2013 for veterinarians [14,15].

The veterinary profession is currently experiencing a shift towards greater awareness and utilisation of EVM approaches [16,17], and this is evident from the increased discussion in the contemporary veterinary literature [18,19]. However, practical training is required to give veterinarians the necessary skills to confidently execute all the components of EVM, particularly if they were not taught these skills as part of their initial veterinary training. In order to practice EVM effectively, practitioners must be able to form a relevant clinical question, know how to correctly use tools to conduct a structured literature search, be able to appraise the literature critically, and finally integrate this information into their clinical decision-making. Vandeweered et al. [20] suggest that the responsibility for EVM should be at three levels; namely education, research (into EVM itself), and with the practitioner. Day one competencies for veterinarians in the UK include elements of EVM [21]; therefore it is likely that veterinary schools in the UK integrate some EVM training into the undergraduate curriculum. However, it is unknown how much training in the principles of EVM is routinely given either at an undergraduate or postgraduate level globally.

There is a need to understand the degree to which EVM is robustly practiced by veterinarians in order to assess the training needs required within the profession. However, before such an assessment is attempted, it is first necessary to ascertain the level of awareness within the veterinary profession of the concept of EVM. The primary aim of this paper is to determine the proportion of veterinarians internationally that are aware of the concept of EVM and to determine whether this differs with location, veterinary training received, and time since graduation. The secondary aim is to investigate how veterinarians have heard of EVM, and a third aim is to ascertain by which methods veterinarians prefer to receive training.

## 2. Materials and Methods

**Figure 1:**
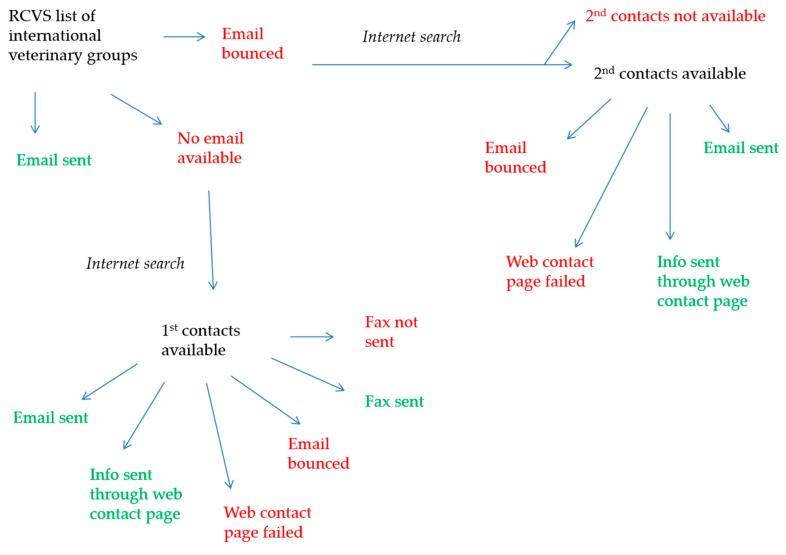
Contacting respondents for the international questionnaire.

The target population was all veterinarians across the globe. A questionnaire on evidence-based veterinary medicine was sent to veterinarians in the UK in 2010/2011 and to other countries in 2011. The questionnaire gained ethical approval from the School of Veterinary Medicine and Science, University of Nottingham (153 100217, UK survey; 395 110615, international survey). The methods of questionnaire dissemination and data collection have been reported previously for the UK survey [22] and for the international survey [23]. There was no definitive global list of veterinarians or organisations available at the time of the study. For respondents working in the UK, the questionnaire was sent to all members listed on the register of the Royal College of Veterinary Surgeons (RCVS) [24] who had agreed for their details to be made available to third party organisations for marketing or research purposes. Respondents of the UK survey received both a paper based questionnaire and a link to access the electronic form of the questionnaire and, thus, the choice of how they submitted their responses. For respondents working outside the UK, veterinary organisations were contacted initially using a list of international veterinary groups from Appendix A of Section 4 of the 2010 RCVS Register of Members. These international veterinary groups were contacted (in the order of preference) by email, via a contact box on a web page, or by fax, using additional searches where necessary (Figure 1). During these additional searches, other organisations of relevance, including managers of online veterinary sites and listserves deemed to be veterinary related, were identified and contacted as described above. Additionally, a snowball sampling approach was also taken; individuals within the Centre for Evidence-based Veterinary Medicine and academic members of staff at the School of Veterinary Medicine and Science at the University of Nottingham were asked to share details for any international veterinary organisations, listserves or chat sites, or personal contacts that they felt might be relevant to this study. These organisations, if not already approached, were contacted directly, or on behalf of the final author (MB). All of contacts outside the UK (with the exception of the online veterinary sites) were emailed a seeding email, followed by the link to the survey between 1.5–3 weeks later, followed by a reminder sent 3–5 weeks after that. Emails were sent between June and September 2011. The questions in the two questionnaires were almost identical, with the main difference being the structure of questions on veterinarians’ use of journals and electronic resources (open questions versus closed questions for the international survey and the UK survey, respectively). The results of veterinarians’ use of journals and electronic resources have previously been published [23,25], and this current paper focusses on veterinarians’ awareness of EVM and their preferred training methods.

Respondents were asked demographic questions and a combination of closed and open questions about EVM and continuing professional development (CPD) training (Supplementary Table S1). The continuing professional development questions were not specific to EVM.

Countries in which veterinarians had trained were assigned to a binary category of developed or developing, according to an adaptation to the intramonetary fund (IMF) classification [26], wherein the countries in the highest income class were classified as developed and those in the remaining four classes were classified as developing [23]. The sources from which respondents had heard of EVM were categorised using an inductive approach in which the main themes observed in the responses were identified, and the responses were assigned to these [27]. This allowed all the responses to be represented and minimised misclassification bias. Where respondents named more than one source, the first source given was used.

Data were sorted in Microsoft Excel (Microsoft Corporation, Redmond, WA, USA, 2007) and descriptive and statistical data analyses performed in Stata 13 (StataCorp, College Station, TX, USA, 2013) [28], with the exception of z-tests for the differences in proportions, which were performed using an online epidemiological tool (Epitools, Ausvet Pty Ltd., Canberra, Australia, 2016) [29].

A binomial logistic regression model was used to investigate the factors associated with whether respondents had heard of EVM. The categories in which respondents had heard of EVM that had a low number of respondents were merged with another appropriate category to eliminate small class sizes. A multinomial logistic regression model was used to investigate factors associated with how respondents had heard of EVM.

To begin with, putative explanatory variables for both models were listed, and factor analysis was used to explore the possible relationships between the explanatory variables [30]. The relationships between the explanatory variables and the outcome variables (Have you heard of EVM (yes/no) and Where did you hear of EVM (Table 3)) were further assessed by means of causal model diagrams, pairwise associations (chi-squared test), and a correlation matrix. A Bonferroni correction [30] was applied to adjust the probability of a Type I error in the case of multiple testing. Where high correlation (>0.7) [31] or multicollinearity (Variance Inflation Factor (VIF) > 5) [32] existed between two variables, the most plausible explanatory variable was retained, the decision for which was aided by the causal model diagram and factor analysis. The remaining variables that showed some association with the outcome variable in univariable analysis (chi-square test, *p* < 0.2) were included in further multivariable analysis. Both models were constructed using manual backwards stepwise regression [30]. Statistical significance was set at *p* < 0.05. For the binomial logistic regression model, model fit was assessed using a Hosmer-Lemeshow goodness-of-fit test, specifying the number of groups for patterns of predictor variables as the largest number less than 10 that would permit sufficiently large expected frequencies (*n* > 5). The predictive ability of the binomial model was assessed by generating a receiver operating characteristics (ROC) curve. For the multinomial logistic regression model, model fit was assessed by performing a Hosmer-Lemeshow goodness-of-fit test adapted for multinomial logistic regression [33], with the specification of number of groups for patterns of predictor variables as per the binomial model. For both models, fit was also assessed by the change in log likelihood with the addition of explanatory variables and a significant likelihood ratio test.

## 3. Results

### 3.1. Respondent Demographics

There were 6310 usable responses analysed in total; 1731 (27.5%) from 68 countries across five continents (Appendix Table A1) in the international survey [23] and 4579 (72.5%) from the UK survey [22]. The majority of respondents to the international survey had heard about the questionnaire via national veterinary associations or boards, or via international or regional veterinary organisations [23].

Of the 6310 respondents, 5384 (85.3%) stated that they were clinicians, 913 (14.5%) stated that they were non-clinicians, and 13 (0.2%) did not state whether they were clinicians or not. The proportion of respondents holding a postgraduate (PG) qualification was lower (*n* = 2549, 40.4%) than those not holding a PG qualification (*n* = 3753, 59.5%; 8 respondents left this question unanswered). Respondents working in developing countries were under-represented (*n* = 146, 2.3%) compared to developed countries (*n* = 5870, 93.0%). It was not possible to determine the development status of the country of work for 294 (4.7%) respondents because these data were not collected from non-clinicians answering the international questionnaire, and a small number of clinicians (*n* = 8) did not declare their country of work or worked in multiple countries (*n* = 5). There were more female (*n* = 3707, 58.7%) than male (*n* = 2576, 40.8%) respondents, although 27 (0.4%) did not declare their gender. The proportion of non-clinicians working outside of the UK (281 of 1731, 16.2%) was significantly higher than non-clinicians working in the UK (632 of 4579, 13.8%, *p* = 0.016, z-value = 2.4). Non-clinicians did a variety of non-clinical work, but those that worked solely in government (*n* = 190, 20.8%), solely in research (*n* = 92, 10.1%), and solely in public health (*n* = 90, 9.9%) formed the majority over any other non-clinical work type or combination of non-clinical work types. More detailed results of respondents’ demographics have previously been reported [22,23].

Factor analysis revealed three main groups, which accounted for the majority of variability within the data. According to the weightings of the contributing variables, these groups were interpreted by the authors as new graduates, UK workers, and those with PG qualifications (Eigenvalues 4.02 and 0.56 and 0.23 respectively). The explanatory variables (*p* < 0.2) that were taken forward for regression analyses were; clinician (yes/no; non-clinician as reference), work in UK (yes/no; work outside the UK as reference), PG or other qualification or degree held (yes/no; no PG qualification as reference), and years since graduation (<8 years, 8–22 years, >22 years; <8 years graduated as reference). The variation inflation factors for these variables were all 1.0. The variable age was excluded due to a high correlation with years graduated (Pearson’s correlation coefficient r = 0.96). A small number of respondents were from developing countries, and this information was only available from clinicians, so country status (developed/developing) was excluded from the model. Gender was excluded because there was a confounding effect of years graduated and being a clinician on gender, and the effect of gender could be rationally explained by these variables.

### 3.2. Respondents That Had Heard of EVM

The vast majority of respondents (*n* = 5420, 85.9%) had heard of the expression “evidence-based veterinary medicine” before participating in the survey (UK survey = 3977, 86.9%; international survey = 1443, 83.4%). The proportion of clinicians from developed countries that had heard of EVM (*n* = 4667 of 5225, 89.3%) was higher than those from developing countries (*n* = 100 of 146, 68.5%) (*p* < 0.0001, z-value = 6.5) (Table 1).

The binomial regression model explained 5% (McFadden’s Pseudo R^2^ = 0.052) of the variance of whether respondents had heard of EVM and correctly classified 64.5% of cases (ROC AUC = 0.645). The model adequately fit the data (Hosmer and Lemeshow analysis) on five quantile groups (quintiles; Χ^2^ = 2.11; *p* = 0.549). Clinicians (OR = 4.00; 95% CI: 3.37, 4.75), respondents working in the UK (OR = 1.32; CI: 1.13, 1.54), and respondents with a PG degree or qualification (OR = 1.77; CI: 1.51, 2.08) were most likely to have heard of EVM (LR Χ^2^ = 264.6, 3 d.f., *p* < 0.0000) (Table 2).

### 3.3. Where Respondents Had Heard of EVM

The most common sources from which respondents had first heard of EVM were vet school or university (*n* = 1207 of 4049, 29.8%); literature (peer-reviewed papers or other publications; hereafter referred to as just “literature”) (*n* = 1074, 26.5%); and CPD conferences, seminars, lectures, and meetings (*n* = 564, 13.9%) (Table 3).

**Table 3:** The most common sources from which respondents had first heard of evidence-based veterinary medicine (EVM).

Where Respondent Heard of EVM	*n*	%
Vet school or university	1207	29.8
Literature (peer-reviewed papers or other publications)	1074	26.5
CPD *, conferences, meetings, seminars or lectures	564	13.9
Postgraduate or specialist study or personal research	158	3.9
Colleague or friend	146	3.6
Work place or I teach it	127	3.1
Emails, e-list, electronic forums, networks or internet	120	3.0
Ubiquitous or various and multiple sources	118	2.9
Don’t know	107	2.6
Another discipline e.g., human medicine or science	99	2.5
General veterinary or professional knowledge or verbal unknown	92	2.3
Veterinary association	92	2.3
Blank or invalid answer	76	1.9
Specialist organisation, research centre or referral clinic	44	1.1
Royal College of Veterinary Surgeons (RCVS) standards or practice audit	10	0.2
Commercial pharmaceutical or nutrition company	8	0.2
Public debate or general media	7	0.2
Total	4049	100

* CPD = Continuing Professional Development.

The three most common sources from which respondents had heard of EVM (vet school or university, literature, CPD) were retained as separate outcome categories for multinomial logistic regression modelling, but those with fewer respondents were merged to form combined classes (Table 4) according to the similarities of outcomes and of significant predictor variables.

The source from which respondents had heard of EVM was associated with working as a clinician, working in the UK, holding a postgraduate qualification, and number of years they had been qualified (*p* < 0.05) (LR = 1569.4, 45 d.f., *p* < 0.05) (Table 5).

The multinomial regression model explained 11.1% (McFadden’s Pseudo R^2^ = 0.111) of the variance in how respondents had heard of EVM and correctly classified 75.0% of cases (ROC AUC = 0.749). The model adequately fit the data (adapted Hosmer and Lemeshow analysis) on six quantile groups (sextiles; Χ^2^ = 24.6; *p* = 0.43). Respondents who had graduated less than 8 years ago were more likely to have heard of EVM via vet school or via PG study than those who had been qualified for longer. Non-clinicians were more likely than clinicians to have heard of EVM via PG study. Clinicians in the UK were more likely than those outside the UK to have heard of EVM via CPD. Respondents working in the UK tended to have had heard of EVM via literature; via the veterinary profession, PG study, vet school; or via CPD. Non-UK respondents were more likely to have heard via email than UK respondents.

There were 6173 responses to whether respondents wanted to find out more about EVM, of which 4256 (68.9%) did. Wanting to find out more about EVM was associated with having heard of EVM, working outside of the UK, being a clinician, having a PG qualification, and having graduated 8–22 years ago (Table 6). The group with the highest proportion that did not want to find out more about EVM were non-clinicians (*n* = 168 of 905, 18.6%). Those that had heard of EVM already (253 of 884, 28.6%) was the group with the highest proportion that said they did not know if they wanted to find out more about EVM (Appendix Table A2).

### 3.4. Further Veterinary Training or Continuing Professional Development

The majority (*n* = 5851 of 6310, 92.7%) of respondents declared that they undertook further training or CPD. For the remaining 459 (7.3%), it was not possible to differentiate between those that did not do any further training from those that left this question unanswered. The majority of both clinicians (*n* = 5082 of 5384, 94.4%) and non-clinicians (*n* = 761 of 913, 83.4%) declared that they did such further training. The proportion of clinicians in developing countries (*n* = 117 of 146, 79.8%) undertaking further training was less than those in developed countries (*n* = 5493, 93.5%) (*p* < 0.0001). The proportion was also less for respondents working outside the UK (*n* = 1556 of 1731, 89.9%) than those working in the UK (*n* = 4295 of 4579, 93.8%; *p* < 0.0001). There was no difference in proportions of those undertaking further training by gender, possession of a postgraduate qualification, or by number of years qualified. Respondents spent a median of 40 h per year (IQR 30–50 h per year) on such training.

The practice or work place paid for at least some of the CPD in 4546 (77.7%) of cases, but it was common for respondents to pay for at least some of their CPD themselves (*n* = 3218, 55.0%). Only 451 (7.7%) respondents had some or all of their CPD paid by “other” means, such as by pharmaceutical companies or from grants.

**Figure 2:**
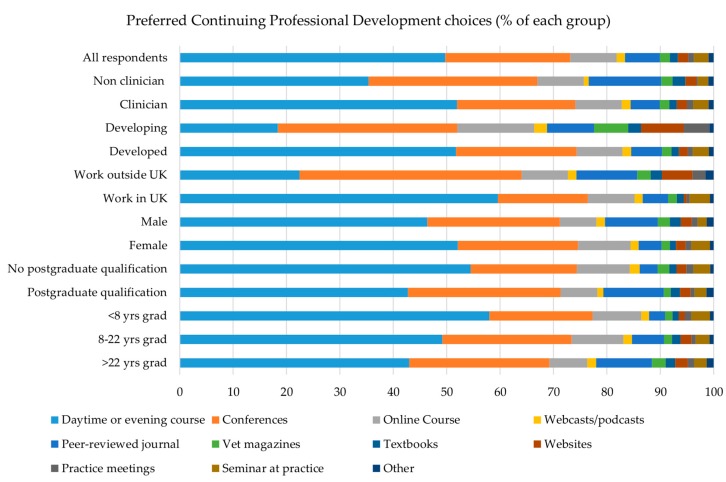
First preferred choice of continuing professional development (CPD) amongst different groups.

Overall the preferred methods of receiving CPD were via day or evening courses (*n* = 2992 of 6017, 49.7%), conferences (*n* = 1409, 23.4%), and online courses (*n* = 524, 8.7%) (Figure 2), and this was also the order of choice for respondents who were clinicians, working in the UK, female, had graduated 22 years ago or less, or had no PG qualification. For respondents who had graduated >22 years ago, who were male or non-clinicians, or had a PG qualification, the first two choices were the same, but peer-reviewed journals were the third most preferred method. Respondents working outside the UK or in developing countries nominated conferences, followed by daytime and evening courses, as their first and second choices, respectively, whilst their third choices were peer-reviewed journals and online courses (Figure 2).

## 4. Discussion

It is encouraging that evidence-based veterinary medicine (EVM) is a term that appears to be well known across the global veterinary profession. Although the vast majority of respondents had heard of EVM, it was a term best known amongst clinicians, those in the UK, and those with a postgraduate (PG) qualification. The number of years since graduation was not associated with whether respondents had heard of the term “evidence-based veterinary medicine”, which contrasts with findings from a study of Belgian veterinarians by Vanderweerd et al. [3]. Vet schools or universities, PG study, and the literature were the main sources from which respondents had heard of EVM. Those that had heard of the term via vet schools or university were mainly newer graduates. This suggests that vet schools are succeeding in introducing the concept to undergraduate veterinary students. Newer graduates were also more likely to have heard the term via PG study, indicating that EVM is being discussed in current veterinary postgraduate training.

Respondents from the UK were more likely than any other group to have heard of EVM via the literature. Around half of the respondents were UK-based, and the vast majority were from developed countries. EVM has been much discussed in both the Veterinary Record and the Journal of the American Veterinary Medical Association in recent years, which have both been identified as the preferred sources of veterinary information for these veterinarians [23,25]. Respondents were not asked to describe what they understood by the term “evidence-based veterinary medicine” or whether they implemented EVM in practice, so further interpretation of respondents’ knowledge about EVM is beyond the scope of this paper.

Continuing professional development in the form of conferences, seminars, and courses was the third most common method of how respondents had heard of EVM. Clinicians were the group most likely to have heard of EVM via CPD, suggesting that this method plays an important role in communicating the concept of EVM to respondents who had not heard of EVM via other sources; for example, those who had not heard via recent vet school or PG programmes. Together with relevant publications in the literature, CPD is an important way of delivering new concepts to all groups of veterinarians working within [34] and outside the UK [35]. However, this survey did not intend to ascertain CPD preferences to receive EVM training in particular but rather CPD as a whole. This survey also showed that preferences on how veterinarians prefer to receive CPD, or similar further training, differ slightly with location and PG training. As a group, those working outside the UK favoured conferences over receiving CPD as a day or evening course. It may be that day and evening courses as a model of CPD delivery are particularly prevalent in the UK so this method was less frequently nominated by this group. However, for those in developing countries particularly, a preference for conferences may be because veterinarians are more sparsely populated and therefore need to travel further to seek training. Time, distance, and the availability of traditional CPD courses therefore make larger and longer conferences a more feasible way of receiving CPD for these groups. Although daytime or evening courses were the second most nominated preferred choice for CPD, rural isolation may explain why online courses were a close third for those in developing countries.

Relatively few respondents had not heard of EVM, and those that were in this group included non-clinicians and those without a PG qualification. Although interaction terms were not used in the regression models, respondents that belong to both of these groups appear to be veterinarians not working in the traditional clinical or research veterinary occupations and, as such, perhaps are unlikely to undertake CPD aimed at clinicians, which may be more likely to include elements of EVM.

More than two-thirds of respondents wanted to hear more about EVM, and this was especially the case for those who had already heard of EVM and those outside the UK. Those who were least likely to want to hear more about EVM were non-clinicians, and this could be because some of this group were working in areas unrelated to clinical practice. Due to the snowball nature of survey dissemination for the international questionnaire, there may also have been a respondent bias towards epidemiologists or those teaching or researching EVM, and they may have been less inclined to say they wanted to find out more about EVM. It is unknown how variable the depth of EVM knowledge amongst those who had heard of EVM was, as respondents were only asked whether they had heard of EVM and not what specific training they had received. However, the appetite of veterinarians across the world for further knowledge is encouraging and suggests there is scope for EVM training. Such training should be available in a form easily accessible to those outside the UK, particularly for those in developing countries. The practice of EVM should be viewed as a career-long endeavour, the key principles and skills of which should be taught in vet schools but with lifelong opportunities for qualified veterinarians to consolidate and further develop EVM skills. The training of undergraduate students in vet schools does indeed appear to be an effective way of communicating the key principles and process of EVM, and training on how to develop critically appraised topics (CATs) has previously been reported as being supported by veterinary students [36]. Training in the key principles and beyond via programmes of CPD and worked examples in the literature would help to fill the gap for those who have not received training and those who embrace the opportunity to test existing EVM skills. Since this survey was conducted, there are initiatives that have been made available to graduated veterinarians; for example, the EBVM Learning tool [37], courses by the CEVM [38] and other organisations, and meetings arranged by RCVS Knowledge [39] and the Evidence-based Veterinary Medicine Association [40].

Given that several years have passed since the collection of these data, it may be that more recent discussions about EVM in the veterinary literature have laid down the foundations for a greater proportion of people to be more receptive to EVM training and CPD. The employment of the principles of EVM in practice may represent something of a cultural shift for certain members of the veterinary profession and potentially necessitate a change in the structure of how a typical day in practice is conducted (for example, allowing sufficient time to keep up to date with the literature). It follows, therefore, that this is not a skill or practice that could be acquired overnight. However, the results of these surveys indicate that there is a willingness across the globe to learn more about EVM and provides teaching groups with information on how EVM training may best be delivered to recipients. Since this survey was conducted, the role of social media in outreach to vets in a professional capacity has increased. The role played by Facebook, Twitter, and the further development and uptake of educational opportunities online were not investigated, and therefore the potential for these media as methods of EVM delivery would be worthy of further investigation in the future.

The authors acknowledge that there were limitations in the study; notably there was a response bias from veterinarians in the U.S. in the international survey and a bias towards respondents in developed countries overall, particularly those from the UK. Since respondents from developing countries were underrepresented in the survey, it is difficult to determine how representative they are of their demographic. Additionally, the method of approaching respondents was not the same for the international and the UK surveys; there was a definitive list of veterinarians registered in the UK via the RCVS register. No such list existed for veterinarians across the globe, so international questionnaire respondents were approached via a method which included a snow ball technique [23]. Respondents were asked to choose preferred CPD choices from a provided list of options rather than nominating their own, which may have influenced the results. There may have been an element of recall bias amongst vets that had been graduated the longest, who may have therefore been less likely to remember specifics from their vet school training, and this may have resulted in an under reporting of hearing of EVM by this group.

## 5. Conclusions

The vast majority of the profession had heard of EVM, and the discipline is being reportedly discussed during undergraduate and postgraduate training and also within the literature, particularly in the UK. Preferences on how CPD is received differ slightly between groups, so this should be borne in mind by EVM training providers when formulating a strategy for dissemination across the profession. Further work is needed to assess veterinarians’ full understanding of EVM, what the barriers are to clinicians using EVM, and how these may be overcome.

## Figures and Tables

**Table 1:** Proportions of respondents that had heard of evidence-based veterinary medicine (EVM) according to several demographic variables.

Group	Heard of EVM		Not Heard of EVM	
	*n*	%	*n*	%
Clinicians (*n* = 5384)	4778	88.7	606	11.3
Non-clinicians (*n* = 913)	633	69.3	280	30.7
Postgraduate qualification (*n* = 2549)	2248	88.2	301	11.8
No postgraduate qualification (*n* = 3753)	3166	84.4	587	15.6
UK (*n* = 4579)	3977	86.9	602	13.1
Non-UK (*n* = 1731)	1443	83.4	288	16.6
Clinician; UK survey (*n* = 3934)	3526	89.6	408	10.4
Clinician; International survey (*n* = 1450)	1252	86.3	198	13.7
Clinician; Developed country * (*n* = 5225)	4667	89.3	558	10.7
Clinician; Developing country * (*n* = 146)	100	68.5	46	31.5

* 13 Clinicians did not declare country of work.

**Table 2:** Characteristics of veterinarians that were most likely to have heard of evidence-based veterinary medicine (EVM) found during multinomial regression.

Respondents Had Heard of EVM	B *	S.E ^†^	z	*p* > |z|	Odds Ratio	95% Confidence Interval	
						Lower	Upper
Clinicians	1.39	0.09	15.9	0.000	4.00	3.37	4.75
Veterinarians working in UK	0.28	0.08	3.45	0.001	1.32	1.13	1.54
Veterinarians with a postgraduate qualification	0.57	0.08	7.09	0.000	1.77	1.51	2.08
Constant	0.30	0.10	2.92	0.004	1.35	1.10	1.64
629 observations				LR chi2(3) = 264.59			
Log likelihood = −2425.7715				Prob > chi2 = 0.0000			

* Coefficient (Beta); ^**†**^ Standard error.

**Table 4:** Outcome variable categories for a multinomial model of where respondents had heard of evidence-based veterinary medicine (EVM).

Where Heard of EVM	*n*	%	Responses Included in Category
Other (reference category)	407	10.1	Don’t know; Ubiquitous; Heard via another (non-veterinary) discipline; Public debate, Blank/invalid
CPD *	564	13.9	CPD, conferences, meetings, seminars or lectures
Email alerts or other email or forum source	120	3.0	Emails, online forums, electronic newsletters
PG **^†^** studies, specialist research or personal interest	285	7.0	PG studies, specialist research or personal interest; Work place or I teach it
General veterinary profession	392	9.7	Veterinary Association, Specialist organization, research centre or referral clinic; Commercial pharmaceutical or nutrition company; General veterinary professional knowledge; Verbal unknown, Colleague or a friend
Literature	1074	26.5	Publications, literature or journals
Vet school	1207	29.8	Vet school
Total	4049	100	

* CPD = Continuing Professional Development; ^**†**^ PG = Postgraduate.

**Table 5:** Multinomial logistic regression model of where respondents had heard of evidence-based veterinary medicine (EVM).

Where Respondents Heard of EVM	B *	S.E ^†^	z	*P* > |z|	Odds Ratio	95% Confidence Interval	
						Lower	Upper
CPD ^**‡**^							
Clinicians	0.54	0.21	2.63	0.008	1.72	1.15	2.57
Veterinarians working in UK	0.30	0.13	2.29	0.022	1.36	1.04	1.76
Veterinarians with PG ^**§**^ qualification	−0.21	0.14	−1.59	0.113	0.81	0.62	1.05
<8 years graduated	Ref						
8–22 years graduated	0.23	0.23	0.99	0.324	1.25	0.80	1.97
>22 years graduated	0.07	0.23	0.32	0.746	1.08	0.69	1.68
Intercept	−0.33	0.30	−1.12	0.263	0.72	0.40	1.28
Email alerts or other email or forum source							
Clinicians	−0.33	0.29	−1.14	0.254	0.72	0.40	1.27
Veterinarians working in UK	−1.70	0.26	−6.50	0.000	0.18	0.11	0.31
Veterinarians with PG ^**§**^ qualification	−0.85	0.23	−3.71	0.000	0.43	0.27	0.67
<8 years graduated	Ref						
8–22 years graduated	0.09	0.36	0.24	0.814	1.09	0.53	2.22
>22 years graduated	0.18	0.36	0.51	0.613	1.20	0.59	2.42
Intercept	−0.12	0.42	−0.29	0.772	0.88	0.38	2.03
PG studies, specialist research or personal interest							
Clinicians	−0.55	0.20	−2.7	0.007	0.58	0.39	0.86
Veterinarians working in UK	0.76	0.17	4.59	0.000	2.14	1.55	2.97
Veterinarians with PG ^**§**^ qualification	0.82	0.17	4.86	0.000	2.27	1.63	3.17
<8 years graduated	Ref						
8–22 years graduated	−0.75	0.24	−3.12	0.002	0.47	0.30	0.76
>22 years graduated	−1.31	0.25	−5.34	0.000	0.27	0.17	0.44
Intercept	0.01	0.31	0.04	0.969	1.01	0.56	1.85
General veterinary profession							
Clinicians	0.03	0.20	0.16	0.872	1.03	0.69	1.54
Veterinarians working in UK	0.73	0.15	4.88	0.000	2.08	1.55	2.78
Veterinarians with PG ^**§**^ qualification	−0.16	0.15	−1.08	0.279	0.85	0.64	1.14
<8 years graduated	Ref						
8–22 years graduated	0.12	0.26	0.47	0.636	1.13	0.68	1.87
>22 years graduated	0.29	0.25	1.15	0.251	1.34	0.81	2.19
Intercept	−0.62	0.32	−1.94	0.052	0.54	0.29	1.01
Literature (peer-reviewed papers and other publications)							
Clinicians	−0.06	0.17	−0.37	0.711	0.94	0.68	1.31
Veterinarians working in UK	0.92	0.12	7.50	0.000	2.51	1.97	3.19
Veterinarians with PG ^**§**^ qualification	−0.31	0.12	−2.56	0.011	0.73	0.57	0.93
<8 years graduated	Ref						
8–22 years graduated	0.21	0.21	1.00	0.315	1.23	0.82	1.85
>22 years graduated	0.20	0.21	0.95	0.341	1.22	0.81	1.82
Intercept	0.42	0.26	1.62	0.106	1.52	0.92	2.51
Vet School or university							
Clinicians	0.23	0.19	1.23	0.218	1.26	0.87	1.82
Veterinarians working in UK	0.38	0.13	2.97	0.003	1.46	1.14	1.87
Veterinarians with PG ^**§**^ qualification	−0.01	0.13	−0.08	0.937	0.99	0.77	1.28
<8 years graduated	Ref						
8–22 years graduated	−2.01	0.19	−10.71	0.000	0.13	0.09	0.19
>22 years graduated	−3.40	0.20	−16.77	0.000	0.03	0.02	0.05
Intercept	2.41	0.25	9.62	0.000	11.17	6.83	18.26
418 observations				LR chi2(30) = 1546.43			
Log likelihood = −6195.7271				Prob > chi2 = 0.0000			

* Coefficient (Beta); ^**†**^ Standard error; ^**‡**^ CPD = Continuing Professional Development; ^**§**^ PG = Postgraduate.

**Table 6:** Characteristics of respondents that wanted to find out more about evidence-based veterinary medicine (EVM).

Respondent Group	*n*	%	Total (Answered Question)	Chi-Square Value	Degrees of Freedom	*p* Value *
Had heard of EVM	3705	70.1	5289	46.3	2	*p* < 0.000
Had not heard of EVM	551	62.3	884			
Work in UK	2940	65.8	4469	367.3	2	*p* < 0.000
Work outside UK	1316	77.2	1704			
Clinicians	3660	69.7	5255	24.6	2	*p* < 0.000
Non-clinicians	589	65.1	905			
PG ^**†**^ qualification	1767	71.2	2481	40.3	2	*p* < 0.000
No PG ^**†**^ qualification	2485	67.5	3684			
<8 years graduated	1198	64.2	1867	53.1	4	*p* < 0.000
8–22 years graduated	1653	73.7	2242			
>22 years graduated	1375	68.0	2021			

* Bonferroni correction adjusted *p* value set at 0.00625 to allow for multiple comparisons; ^**†**^ PG = postgraduate.

## Supplementary Materials

The following are available online at www.mdpi.com/2306-7381/4/1/15/s1, SpreadsheetS1_: SpreadsheetS1_EVM dataset _Huntley et al.xlsx, SupplementarydocS2_: Table S1_Open and closed questions in evidence-based medicine section of questionnaire.docx.

## Appendix A

**Table A1:** Continents and sub-continents of respondents.

Africa		Americas		Asia	
Eastern Africa	3	Caribbean	25	Eastern and South-Eastern Asia	23
Middle and Southern Africa	87	Central America	3	Southern Asia	22
Northern Africa	5	North America	606	Western Asia	9
Western Africa	7	South America	7		
Europe		Oceania		Unknown	
Eastern Europe	3	Australia and New Zealand, Melanesia and Polynesia	154	Country not stated	289
Northern Europe	4848			Multiple countries	5
Southern Europe	47				
Western Europe	167				

**Table A2:** Responses of interest to finding out more about evidence-based veterinary medicine (EVM).

Would Like to Find Out More about EVM							
Group	Yes	%	No	%	Don’t Know	%	Subtotal
All	4256	68.9	868	14.1	1049	17.0	6173
Clinicians	3660	69.6	697	13.3	898	17.1	5255
Non-clinicians	589	65.1	168	18.6	148	16.4	905
Non-developed	129	89.6	8	5.6	7	4.9	144
Developed	3912	68.1	833	14.5	996	17.3	5741
Work in UK	1316	77.2	156	9.2	232	13.6	1704
Work outside UK	2940	65.8	712	15.9	817	18.3	4469
No PG ***** qualification	2485	67.5	485	13.2	714	19.4	3684
PG qualification	1767	71.2	380	15.3	334	13.5	2481
Male	1808	71.6	392	15.5	324	12.8	2524
Female	2434	67.1	470	13.0	722	19.9	3626
<8 years graduated	1198	64.2	300	16.1	369	19.8	1867
8–22 years graduated	1653	73.7	243	10.8	346	15.4	2242
>22 years graduated	1375	68.0	317	15.7	329	16.3	2021
Heard of EVM	551	62.3	80	9.0	253	28.6	884
Not heard of EVM	3705	70.1	788	14.9	796	15.1	5289

* PG = Postgraduate.
