# Supplementary material for: The Awareness of the International Veterinary Profession of Evidence-Based Veterinary Medicine and Preferred Methods of Training
Source: Vet Sci. 2017 Mar 8;4(1):15. doi: 10.3390/vetsci4010015 (PMC5606622; doi:10.3390/vetsci4010015)
Supplement: Supplementary file 1 [file vetsci-04-00015-s001.pdf]

Supplementary Materials: The Awareness of the International Veterinary Profession of Evidence-Based Veterinary Medicine and Preferred Methods of Training

Selene J. Huntley, Rachel S. Dean and Marnie L. Brennan

Table S1. Open and closed questions in evidence-based medicine section of questionnaire.

Questions Asked	Type of Question	Response Options
Evidence-based Veterinary Medicine Section		
Before participating in this survey, had you heard of the expression "evidence-based veterinary medicine"?	Closed	2 options given
Where did you hear about it?	Open	
Would you be interested in finding out more about evidence-based veterinary medicine?	Closed	3 options given
Sources of Information or Evidence		
Do you undertaken any further training for the veterinary work you do, e.g., attending conferences? *	Closed	2 options given
Approximately how many hours of further training do you do every year?	Open	
Who pays/finances your training?	Closed, with an "Other" section	3 options given (including "Other")
Please indicate which of the following further training methods you would choose as your preferred first, second and third choice	Closed, with an "Other" section	9 options given (including "Other")

* International questionnaire only; such training is mandatory for veterinarians working in the UK.

© 2017 by the authors; licensee MDPI, Basel, Switzerland. This article is an open access article distributed under the terms and conditions of the Creative Commons by Attribution (CC-BY) license (http://creativecommons.org/licenses/by/4.0/).
